# Neuromuscular effects of G93A-SOD1 expression in zebrafish

**DOI:** 10.1186/1750-1326-7-44

**Published:** 2012-08-31

**Authors:** Stacey A Sakowski, J Simon Lunn, Angela S Busta, Sang Su Oh, Grettel Zamora-Berridi, Madeline Palmer, Andrew A Rosenberg, Stephen G Philip, James J Dowling, Eva L Feldman

**Affiliations:** 1 Department of Neurology, University of Michigan, 109 Zina Pitcher Place, Ann Arbor, 5017 AAT-BSRB, MI, USA; 2 Department of Pediatrics & Communicable Diseases, University of Michigan, 109 Zina Pitcher Place, Ann Arbor, 5019 AAT-BSRB, MI, USA

**Keywords:** Amyotrophic lateral sclerosis (ALS), Motor neuron (MN), Zebrafish, Cu^2+^/Zn^2+^ superoxide dismutase (SOD1), G93A-SOD1, Neuromuscular junction, Neurodegeneration

## Abstract

**Background:**

Amyotrophic lateral sclerosis (ALS) is a fatal disorder involving the degeneration and loss of motor neurons. The mechanisms of motor neuron loss in ALS are unknown and there are no effective treatments. Defects in the distal axon and at the neuromuscular junction are early events in the disease course, and zebrafish provide a promising in vivo system to examine cellular mechanisms and treatments for these events in ALS pathogenesis.

**Results:**

We demonstrate that transient genetic manipulation of zebrafish to express G93A-*SOD1*, a mutation associated with familial ALS, results in early defects in motor neuron outgrowth and axonal branching. This is consistent with previous reports on motor neuron axonal defects associated with familial ALS genes following knockdown or mutant protein overexpression. We also demonstrate that upregulation of growth factor signaling is capable of rescuing these early defects, validating the potential of the model for therapeutic discovery. We generated stable transgenic zebrafish lines expressing G93A-*SOD1* to further characterize the consequences of G93A-*SOD1* expression on neuromuscular pathology and disease progression. Behavioral monitoring reveals evidence of motor dysfunction and decreased activity in transgenic ALS zebrafish. Examination of neuromuscular and neuronal pathology throughout the disease course reveals a loss of neuromuscular junctions and alterations in motor neuron innervations patterns with disease progression. Finally, motor neuron cell loss is evident later in the disease.

**Conclusions:**

This sequence of events reflects the stepwise mechanisms of degeneration in ALS, and provides a novel model for mechanistic discovery and therapeutic development for neuromuscular degeneration in ALS.

## Background

Amyotrophic lateral sclerosis (ALS) is an adult-onset, relentlessly progressive, and ultimately lethal neuromuscular disease involving the degeneration and loss of motor neurons (MNs). Effective treatments for ALS have been elusive due to the fact that the mechanisms of MN loss in ALS are unknown. It is likely that multiple cell types within the MN microenvironment and multiple mechanisms contribute to ALS pathogenesis [1,2]. While MN death is associated with caspase-mediated programmed cell death, neuromuscular junction (NMJ) defects and axonal degeneration are evident early in the disease course before symptom onset and MN loss [3,4]. Understanding the causes of NMJ and axonal degeneration in ALS and identifying therapies that protect against these early insults represent the best strategy for preventing the irreversible loss of MNs.

The majority of ALS is sporadic with unknown etiology (90-95%); however, the remaining cases are familial (fALS) and have been linked to genetic mutations in genes such as Cu^2+^/Zn^2+^ superoxide dismutase (*SOD1*), Alsin (*Als2*), dynactin, angiogenin, senataxin, vesicle-associated membrane protein/synaptobrevin-associated membrane protein B, TAR DNA binding protein 43 (*TDP43*), fused in sarcoma (*FUS*), optineurin, and ubiquilin 2, or a hexanucleotide repeat expansion in *C9ORF72*[5-18]. *SOD1* mutations are associated with 10-20% of fALS cases and currently more than 150 missense mutations in *SOD1* are reported, all of which are associated with a toxic gain of unknown function. Many in vitro and in vivo models expressing mutant SOD1 have been developed to study disease pathogenesis [2,19]. G93A-*SOD1* is the most thoroughly characterized ALS mutation and expression in primary MN cultures results in caspase activation and MN death [20,21]. In vivo ALS models, including transgenic G93A-*SOD1* mice, exhibit MN degeneration, muscle weakness and early lethality, resembling the human ALS disease course [22,23]. G93A-*SOD1* mice also show denervation at NMJs, defects in axonal transport, and axonal degeneration well before the onset of clinical symptoms, which supports a “dying-back” mechanism of MN degeneration [24,25]. Thus, understanding and targeting the distal degeneration that occurs prior to the irreversible loss of MNs is imperative for developing effective therapies for ALS.

Recently, zebrafish have been gaining momentum as an emerging technology for the study of neurodegenerative diseases [26,27]. Zebrafish are ideal for the development of novel strategies to understand ALS pathogenesis and screen potential therapies due to the fact that they develop quickly, have large numbers of offspring, are less expensive than rodent models, and they are easily amenable to genetic manipulation. Zebrafish neuromuscular physiology is also similar to humans, neuromuscular development is well characterized and rapid, and many tools exist to closely examine MN and muscle pathology in zebrafish. Furthermore, mechanistic insight into ALS pathogenesis could also be possible through combination with various tissue- and organelle-specific transgenic reporter zebrafish lines available [28-31], and some neurodegenerative disease models have already been developed. Spinal muscular atrophy, a neurodegenerative disease involving MNs, has been successfully modeled in zebrafish [32-34], and transient expression of select ALS mutations has also been reported. Transient overexpression of mutant SOD1 or mutant FUS induces an axonopathy phenotype at 30 hours post-fertilization (hpf) characterized by decreased axon length and aberrant branching [35,36], as does knockdown of *fus*, *als2*, and *elp3*[35,37,38]. Similarly, knockdown of *tdp43* or expression of mutant *TDP43* in zebrafish embryos results in an axonal phenotype early in development, which is further evidenced in swimming deficits at 48 hpf [39]. While these results reflect an effect of mutant ALS proteins on developing MN axons, the transient expression in these models limits the amount of insight that can be gained regarding the mechanisms of progressive neurodegeneration that is characteristic of ALS.

To investigate ALS disease pathogenesis and progression in zebrafish, we generated novel stable transgenic zebrafish ubiquitously expressing G93A-*SOD1*. At the onset of this project, no transgenic ALS zebrafish models were established to our knowledge; however, in 2010 the Beattie laboratory reported the generation and characterization of transgenic zebrafish lines ubiquitously expressing zebrafish G93R-*sod1*[40]. This model exhibits motor deficits, MN degeneration and loss, and effects on survival [40] that are characteristic of ALS, validating our approach to generate and utilize the transgenic ALS zebrafish model described in this report. Our initial experiments verified the potential of human G93A-*SOD1* to induce the previously reported motor axon defects, and we also demonstrated attenuation of these MN axonal defects with a known neuroprotective factor, insulin-like growth factor-I (IGF-I). We then established stable transgenic zebrafish lines expressing GFP-tagged human G93A-*SOD1* using Tol2-mediated transgenesis. Characterization of our stable transgenic G93A-*SOD1*-GFP zebrafish revealed motor deficits that coincide with defects at the NMJ and subsequent alterations in neuromuscular innervation. We also observe MN loss later in the disease course, which supports a sequence of events that resemble human ALS pathogenesis. Overall, this report describes our detailed examination of MN axonal pathology, NMJ integrity, and MN loss in the context of disease onset and progression in a novel in vivo zebrafish ALS model that will be instrumental in future studies of ALS disease mechanisms and therapeutic development.

## Results

### Transient overexpression of G93A-SOD1 in zebrafish causes motor axon defects

**Figure 1:**
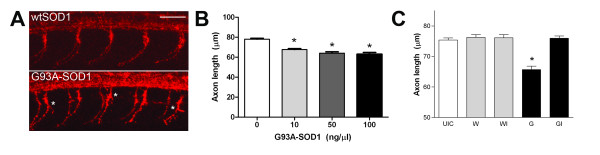
**Effects of G93A-*SOD1*on developing zebrafish MN axons.** Zebrafish embryos at the 1-4 cell stage were microinjected with wt*SOD1*, G93A-*SOD1* and/or *igf-I* RNA after timed mating of adult AB strain zebrafish and underwent IHC with the SV2 antibody at 30 hpf to visualize MN axons. (**A**) SV2 IHC demonstrates increased branching of motor axons, represented by (*) in embryos injected with G93A-*SOD1* (50 ng/μl) RNA, compared to embryos injected with wt*SOD1* (50 ng/μl) RNA. 20x magnification confocal images; scale bar equals 50 μm. (**B**) Measurement of axon length in embryos injected with 0-100 ng/μl G93A-*SOD1* RNA reveals a dose-dependent decrease in MN axon length; * P < 0.001 compared to UIC. (**C**) Co-injection of *igf-I* RNA (I; 200 ng/μl) attenuates the effects of G93A-*SOD1* (G; 50 ng/μl) on axon length. No significant effects of wt*SOD1* (W; 50 ng/μl) are observed, regardless of IGF-I expression. * P < 0.001 compared to UIC.

Before generating stable transgenic G93A-*SOD1* zebrafish, we first examined developing zebrafish MN axons following transient human G93A-SOD1 expression. Microinjection of G93A-*SOD1* RNA (0 – 100 ng/μl), which was generated from a pCS2 vector that enables efficient expression in zebrafish, results in transient overexpression of the protein as confirmed by western blotting (data not shown). SV2 immunohistochemistry (IHC) reveals that G93A-SOD1 expression results in abnormal premature branching of motor axons at 30 hpf compared to controls (Figure 1A, *). We also detect a dose-dependent effect of G93A-SOD1 expression on axon length compared to uninjected control (UIC) embryos, with significant decreases observed at concentrations as low as 10 ng/μl (Figure 1B). Specifically, axon length is decreased by 13.2%, 17.9%, and 19.1% at doses of 10, 50, and 100 ng/μl G93A-*SOD1* RNA, respectively. Co-injection of G93A-*SOD1* RNA (50 ng/μl) with *igf-I* RNA (200 ng/μl) demonstrates that these defects are attenuated by IGF-I upregulation (Figure 1C), a known neuroprotective strategy in ALS model systems [41]. Wild-type SOD1 (wtSOD1) expression has no effect on axon length (Figure 1C). IGF-I upregulation was confirmed by assessing downstream activation of Akt and p44/42 mitogen activated protein kinase (MAPK) signaling and SOD1 expression was confirmed for all clutches using western blotting (data not shown). Together, these findings demonstrate that expression of mutant human SOD1 in zebrafish results in a motor axon phenotype that is amenable to therapeutic intervention, validating the potential usefulness of zebrafish as a model for investigating ALS pathogenesis and drug discovery.

### Generation of stable transgenic G93A-SOD1 zebrafish lines

**Figure 2:**
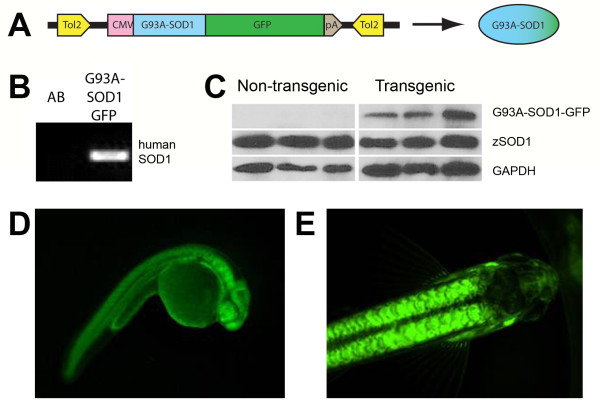
**Transgenic ALS zebrafish.** Transgenic ALS zebrafish are generated by Tol2-mediated transgenesis. (**A**) Constructs include a 5’ CMV promoter, human G93A-SOD1 and a 3’ EGFP C-terminal fusion protein including a SV40 late poly A tail. Microinjection of construct cDNA in the presence of *Tol2 transposase* RNA facilitates efficient, random integration of the transgene via flanking Tol2 sites. (**B**) PCR of genomic DNA extracted from 24 hpf F1 generation embryos demonstrates germline transmission of the G93A-*SOD1*-GFP transgene, confirming successful generation of the transgenic lines. (**C**) Western immunoblotting of protein lysates from 24 hpf F1 generation embryos demonstrates expression levels of the G93A-*SOD1*-GFP protein in three independent clutches of transgenic embryos. (**D**-**E**) Images of an F1 embryo (D; 24 hpf) and adult (E; 3 months of age) from the G21 G93A-*SOD1*-GFP transgenic line demonstrate stable ubiquitous expression of the fusion protein throughout the zebrafish lifespan.

Stable transgenic ALS zebrafish lines were successfully generated for these studies using Tol2-mediated transgenesis. Coinjection of pDEST-CMV-G93A-*SOD1*-GFP cDNA (Figure 2A), in combination with *Tol2 transposase* RNA, resulted in random insertion of the Tol2-flanked transgene into the zebrafish genome. Preliminary studies comparing G93A-*SOD1* and G93A-*SOD1*-GFP RNA injections did not demonstrate an effect of utilizing a GFP-fusion construct on the MN axonal phenotype (data not shown). Germline transmission was confirmed by PCR analysis of genomic DNA from F1 generation embryos for the human transgene (Figure 2B). Western blotting of protein lysates from F1 generation embryos for SOD1 expression (Figure 2C) demonstrated that G93A-SOD1-GFP expression levels were approximately 64% that of the endogenous zebrafish wtSOD1. Transgenic lines expressed the GFP-fusion protein ubiquitously throughout the lifespan of the zebrafish (Figure 2D-E). F2 generation zebrafish from 2 independent lines were characterized for these studies, the G93A-*SOD1*-GFP.umich1 (G1) and the G93A-*SOD1*-GFP.umich21 (G21) lines, to minimize the chance of any positional effects of the insertions. All presented data is for the G21 line, as both lines exhibited similar morphological phenotypes.

### Symptom onset in transgenic G93A-SOD1 zebrafish

**Figure 3:**
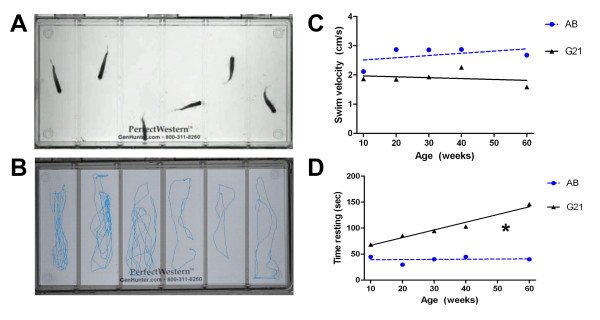
**Activity monitoring in transgenic ALS zebrafish.** (**A**): Representative experimental layout for monitoring spontaneous swimming behavior of 20 week old control (wells 1-3) and transgenic G93A-*SOD1*-GFP (wells 4-6) zebrafish using the Noldus Larvae Activity Monitoring System. (**B**) Movement tracks extrapolated from a 1 minute trial by EthoVision for analysis of multiple different activity parameters. (**C**) While minimal differences are observed in the spontaneous activity levels of transgenic and control zebrafish throughout the disease course, transgenic G93A-*SOD1*-GFP zebrafish exhibit a consistently lower swimming velocity relative to age-matched AB controls. (**D**) As the disease progresses, transgenic G93A-*SOD1*-GFP zebrafish spend more time resting than age-matched control AB zebrafish. * P < 0.01 compared to AB control slope.

Disease onset in non-transgenic AB control or transgenic G93A-*SOD1*-GFP zebrafish was determined by quantitative assessment of spontaneous swimming behavior using the Noldus Activity Monitoring System as a clinical correlate to extremity weakness observed in ALS patients (Figure 3). The system integrates movements over a defined interval for various activity parameters, including swimming velocity and time spent swimming versus resting. Examination of spontaneous swim speeds demonstrate that, while the swimming velocity at 10 weeks of age is similar for control and transgenic zebrafish lines, the transgenic G93A-*SOD1*-GFP zebrafish exhibit an overall slower velocity throughout the disease course compared to AB control zebrafish (Figure 3C). Transgenic G93A-*SOD1*-GFP zebrafish also tend to spend more time resting as the disease progresses (Figure 3D). Linear regression analysis, on the other hand, reveals that the overall change in swimming velocity between 10-60 weeks of age is approximately zero for both lines; slopes equal 0.0076 and -0.0889 for control AB and transgenic G21 zebrafish, respectively (Figure 3C). This could reflect variable onset among the transgenic G93A-*SOD1*-GFP zebrafish line that does not reach significance, or may indicate that significant differences in progression might occur later than the 60 week time point. Analysis of the overall change in time resting, however, is significantly different between 10-60 weeks of age for the two lines (Figure 3D). Taken together, these swimming behaviors represent motor dysfunction and decreased endurance that are consistent with a neuromuscular disease, and reflect the eventual onset and progression of disease in transgenic ALS zebrafish.

### NMJ and axon pathology in transgenic G93A-SOD1 zebrafish

**Figure 4:**
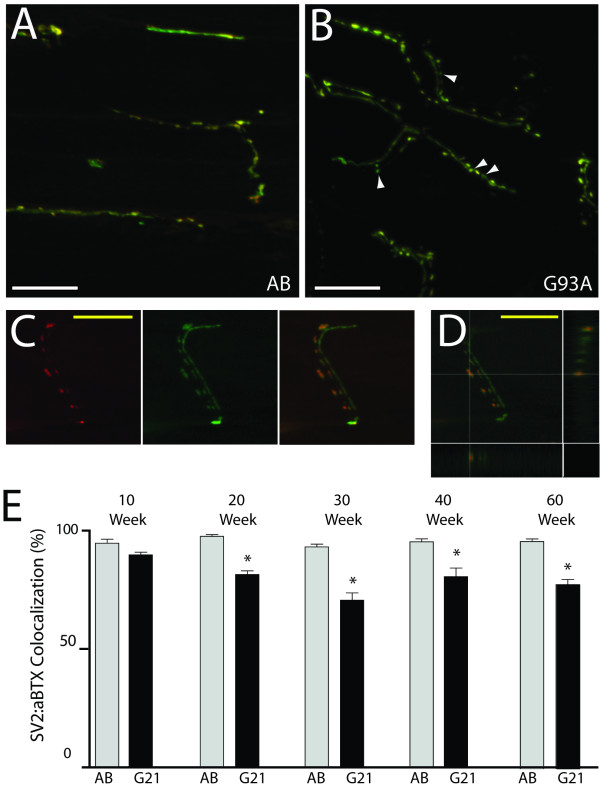
**Neuromuscular junction integrity in transgenic ALS zebrafish.** NMJs in transverse muscle sections from control AB and transgenic G93A-*SOD1*-GFP zebrafish were examined at multiple timepoints after αBTX staining (red) to label AChR clusters in the muscle fibers and SV2/NF IHC (green) to label MNs. Colocalization (yellow) is apparent at intact NMJs. (**A**-**B**) Representative control AB zebrafish (A) exhibit long axons with multiple synapses, whereas transgenic G93A-*SOD1*-GFP zebrafish (B) exhibit shorter axons and varied innervation patterns with some denervated NMJs (arrowheads). 60x oil magnification confocal images; scale bar = 20 μm. (**C**-**D**) A representative image of an individual neuron, with images included as both separated and merged fluorescent channels (C) and visualized with orthogonal XZ and YZ views of the z-series confocal image (D), validates that colocalization of SV2/NF and αBTX is representative of NMJs. 60x oil magnification confocal images; scale bar = 20 μm. (**E**) Quantification of SV2/NF:αBTX colocalization in 10-60 week old control AB and transgenic G93A-*SOD1*-GFP zebrafish. * P < 0.0001 compared to age-matched AB controls.

**Figure 5:**
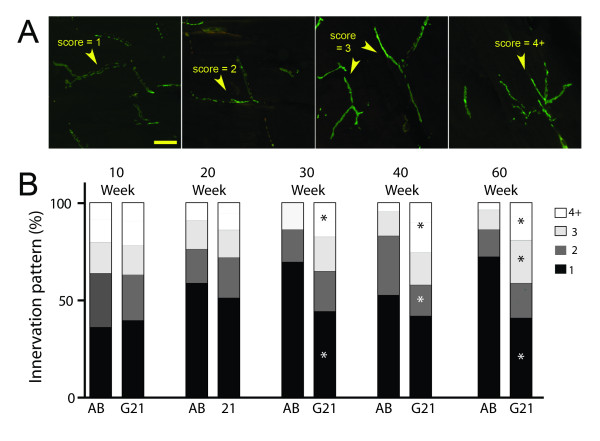
**Neuromuscular innervation characterization in transgenic ALS zebrafish.** Analysis of innervation patterns in 10-60 week old control AB and transgenic G93A-*SOD1*-GFP zebrafish. (**A**) Axons are scored as long (score = 1), moderately branched (scores = 2 or 3) or complex (score = 4+), as demonstrated in representative images. (**B**) Quantification of scoring results in 10-60 week old control AB and transgenic G93A-*SOD1*-GFP zebrafish. * P < 0.01 compared to age-matched AB controls.

Effects at the distal axon and NMJ are one of the earliest defects in ALS [3,4,24,25]; therefore, we next investigated neuromuscular pathology in AB control and transgenic G93A-*SOD1*-GFP zebrafish. NMJ integrity was first examined by quantifying the percentage of colocalized postsynaptic α-bungarotoxin (αBTX)-positive acetylcholine receptor (AChR) clusters and SV2/neurofilament (NF)-positive MN axons (Figure 4A-D; arrowheads depict defects at NMJs). At 10 weeks of age, no differences were observed between control and transgenic G93A-*SOD1*-GFP zebrafish; however, transgenic G93A-*SOD1*-GFP zebrafish exhibited a significant loss of intact NMJs after 20 weeks of age that persisted through the 60 week time point (Figure 4E). We next examined how MNs innervated the muscle fibers by qualitatively scoring the innervation patterns at each time point as long, moderately branched, or complex (Figure 5). Control AB zebrafish predominantly exhibited long or moderately branched axons at all examined time points (Figure 4A and Figure 5B), whereas the percentage of complex branched fibers increased around 30 weeks of age in the transgenic G93A-*SOD1*-GFP zebrafish (Figure 4B and Figure 5B). Together, these data reveal disruption at the NMJ with disease progression in transgenic G93A-*SOD1*-GFP zebrafish that is followed by alterations in MN innervation patterns that may reflect compensatory remodeling of NMJs.

### MN loss in transgenic G93A-SOD1 zebrafish

**Figure 6:**
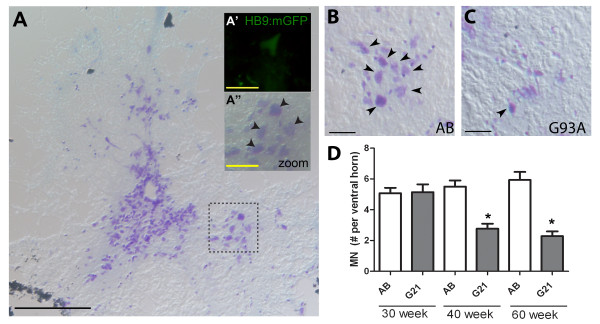
**MN loss in transgenic ALS zebrafish.** Spinal cord cross sections from 30-60 week old zebrafish were stained with Cresyl violet Nissl (purple) to label MNs. (**A**) Representative low power images from control AB zebrafish is provided for orientation purposes; scale bar = 200 μm. Inset zoomed images of the spinal cord ventral horn from AB (A”) and transgenic HB9:mGFP (A’) zebrafish validate our approach to quantify MN number based on cell body size and localization to the ventral horn; scale bar = 25 μm. (**B**-**C**) Representative images from control AB (B) and transgenic G93A-*SOD1*-GFP (C) zebrafish at 60 weeks of age. Arrowheads denote motor neurons; 20X magnification images; scale bar = 100 μm. (**D**) Quantification of MN counts per ventral horn in 30, 40 and 60 week old control AB and transgenic G93A-*SOD1*-GFP zebrafish. * P < 0.0001 compared to age-matched AB controls.

Axonal and NMJ degeneration is followed by MN loss in ALS patients and transgenic ALS rodents [3,42], prompting us to next examine MN numbers in the spinal cord of transgenic G93A-*SOD1*-GFP zebrafish. Spinal cord cross-sections from control AB and transgenic G93A-*SOD1*-GFP zebrafish were labeled with Cresyl violet Nissl stain and MN loss was evaluated by quantifying the number of large MN cell bodies per ventral horn (Figure 6B-C, arrowheads), an approach validated using transgenic HB9:mGFP zebrafish [28] which express GFP under control of the MN-specific HB9 promoter (Figure 6A). Control AB zebrafish exhibited a similar number of MNs per ventral horn at every time point examined, whereas transgenic G93A-*SOD1*-GFP zebrafish exhibited an approximate 50% loss in MN number by 30 weeks of age (Figure 6D). Overall, our findings indicate that G93A-*SOD1*-GFP expression results in alterations in swimming behavior, NMJ denervation and subsequent effects on NMJ innervation patterns which coincide with MN degeneration and loss.

## Discussion

ALS is a relentlessly progressive neuromuscular disease with no effective treatments. While the cause of MN degeneration in ALS is unknown, multiple lines of evidence support a “dying-back” phenomenon that is characterized by defects at the NMJ and in distal MN axons which is then followed by subsequent MN loss [3,4,24,42]. In vitro studies demonstrate protective effects of caspase inhibition and growth factor therapies on MN survival [21,41]. However, studies in in vivo models indicate that treatments targeted at MN loss have limited, if any, impact on disease symptoms or survival [20,43,44] and that neuroprotective strategies targeted at muscle and NMJs such as growth factor therapies improve symptom onset and severity [24,41,45,46]. Clinical translation of such strategies, however, has not yet been realized and improved treatments for ALS are required. Because defects in distal axons and at NMJs occur before symptom onset and MN loss in ALS, comprehending the mechanisms of ALS neuromuscular pathogenesis is essential for the development of therapies targeted at events prior to the irreversible loss of MNs. Detailed characterization of neuromuscular morphological alterations is possible in zebrafish; therefore, we investigated the effects of G93A-SOD1 expression on zebrafish MNs and NMJs.

In recent years, a handful of studies have demonstrated the consequences of transient expression or knockdown of select ALS mutant proteins in zebrafish embryos [35-39]. Expression of mutant forms of SOD1, for example, induce early defects in MNs including decreased axon length and aberrant branching at 30 hpf, which are attenuated or exacerbated by vascular endothelial growth factor upregulation or knockdown, respectively [36]. Therefore, we first examined the effects of transient human G93A-*SOD1* on zebrafish embryo MNs. Because RNA was generated from a pCS2-based construct known to elicit efficient high-level expression in zebrafish, we utilized a dose range that was between 10- to 100-fold lower than the previous report. As seen in Figure 1, our findings confirm the previous axonopathy findings, and further demonstrate that significant effects can be observed even with low levels of G93A-*SOD1* RNA. We then examined the therapeutic efficacy of IGF-I upregulation by co-injecting *igf-I* RNA with G93A-*SOD1* RNA and assessing axonal development. Despite the fact that clinical translation of IGF-I therapies for ALS have been slow and not yielded the expected outcomes, IGF-I has been extensively studied for ALS. In vitro and in vivo studies demonstrate that IGF-I activates neuroprotective signaling pathways and provides protection to MNs and at NMJs [41]. Along those lines, IGF-I is capable of preventing the decreased axon length observed with G93A-SOD1 expression in zebrafish embryos (Figure 1C). Together with the previous report [36], these data demonstrate an effect of low levels of human G93A-*SOD1* on zebrafish MNs that is amenable to neuroprotective intervention, providing rationale for the generation of stable transgenic G93A-*SOD1* zebrafish lines to examine neuromuscular pathogenesis in ALS.

Zebrafish are easily amenable to genetic manipulation and multiple approaches exist for the generation of stable transgenic disease models. The transgenic zebrafish lines described in this study (Figure 2) were developed using Tol2-mediated transgenesis. Briefly, constructs were generated by multisite Gateway cloning which provides a simple approach to generate Tol2-flanked constructs with various promoters and fusion elements that are amenable to random genomic insertion in the presence of transposase [47]. The CMV promoter we used is derived from the pCS2+ vector used in our transient studies, and a C-terminal GFP-fusion allows for easy detection of germline transmission and confirmation of cellular expression patterns. Confirmation that utilization of a GFP-fusion construct does note exacerbate the phenotype was supported by preliminary studies comparing transient expression of G93A-SOD1 and G93A-SOD1-GFP in embryos (data not shown). Other reports also demonstrate that GFP expression does not affect the mutant SOD1 phenotype in embryos [36], and the use of GFP in transgenic tissue-specific reporter zebrafish lines like HB9:mGFP zebrafish that express GFP in MNs [28] provide further evidence supporting our approach. The Tol2-based transposon system supports dramatically increased transgenesis efficiencies [48], and using this approach, we were able to establish 6 independent transgenic G93A-*SOD1*-GFP zebrafish lines. The G1 and G21 lines characterized for these studies exhibit ubiquitous expression of the transgene that persists throughout the zebrafish lifespan and through multiple generations.

Transgenic zebrafish are an emerging model for the study of neurodegenerative diseases and at the initiation of this project, long-term data was not available on how chronic G93A-SOD1 expression, or late-onset neurodegeneration in general, would affect zebrafish. Therefore, we first examined swimming behavior as a correlate to muscle weakness observed in ALS patients using the Noldus Larvae Activity Monitoring System. Spontaneous swimming behavior was regularly monitored for quantification of swim speed and duration of movement during a defined interval. Linear regression analysis of AB control and transgenic G93A-*SOD1*-GFP zebrafish swimming velocity between 10 and 60 weeks of age (Figure 3C) did not reveal a significant difference in overall swim speed change, or slope, between the 2 lines. What is important to note, however, is that examination of the individual time points reveals similar swim speeds at 10 weeks of age, whereas apparent differences in the degree of movement emerge after 20 weeks of age between the 2 lines, with the transgenic G93A-*SOD1*-GFP zebrafish consistently exhibiting a trend of slightly slower swim speeds. Transgenic G93A-*SOD1*-GFP zebrafish, however, do tend to spend more time resting at advanced time points compared to controls (Figure 3D). These findings may indicate that, although there are no striking changes in locomotor potential between 10-60 weeks of age in either line, the increased time resting could reflect a higher tendency for fatigue in the transgenic G93A-*SOD1*-GFP zebrafish that may precede and potentially predict later motor phenotypes. Given the drastic motor deficits that appear in rodent ALS models [22,49], these modest effects were initially surprising, but during the course of these studies the Beattie laboratory reported the first generation and characterization of a transgenic ALS zebrafish model [40]. In that study, the transgenic zebrafish overexpress zebrafish G93R-sod1 and exhibit decreased swimming endurance at 12 and 16 mo of age compared to non-transgenic controls, as measured by calculating critical swimming speeds against an increasing current flow [40]. These findings suggest that more drastic effects on movement behavior in our model may not be apparent within the 60 week time frame examined and support the continued evaluation of swimming behavior in our transgenic lines, and also suggest the possibility that muscle strength defects in our model may become apparent by exploring additional assays for motor function.

**Figure 7:**
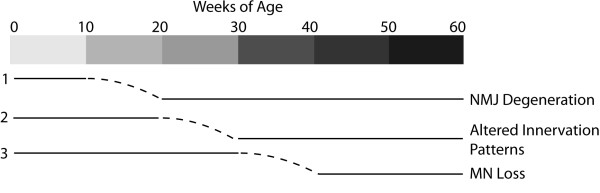
**Neuromuscular phenotype of transgenic ALS zebrafish.** Transgenic G93A-*SOD1*-GFP zebrafish exhibit denervation at the NMJ around 20 weeks of age, subsequent alterations in innervation patterns at 30 weeks of age, and evidence of MN loss around 40 weeks of age which reflect a sequence of events that resembles the human disease course.

As previously described, neuromuscular defects are apparent in ALS rodent models and patients well before symptom onset and MN loss [3,4]; therefore, we next performed a detailed pathological characterization of NMJs and MNs in control and transgenic G93A-SOD1-GFP zebrafish. Techniques to quantify NMJ innervation by MNs are well-established in rodents [25,45,50,51] and larval zebrafish [52-55], however, only limited information regarding visualization and quantification of juvenile and adult zebrafish NMJ integrity are available [32,56]. For the current studies, we labeled presynaptic MNs and postsynaptic AChR clusters with SV2/NF and αBTX, respectively, and quantified the number of intact NMJs in control AB and transgenic G93A-*SOD1*-GFP zebrafish between 10 and 60 weeks of age (Figure 4). Transgenic G93A-*SOD1*-GFP zebrafish exhibited a significant loss of intact NMJs at 20 weeks of age that was consistent across the remaining time points. These effects at the NMJ even in the absence of significant symptomatic affects are consistent with those observed in rodent ALS models. Transgenic G93A-*SOD1* mice exhibit a 40% reduction in the number of innervated NMJs at 47 days of age, while symptomatic onset does not occur until around 80 days of age [3]. The importance of NMJ maintenance is further supported by a study where muscle-specific expression of mutant SOD1 in mice promoted NMJ defects and distal axonal degeneration [57]. Interestingly, we noticed that this early decrease in NMJ integrity at 20 weeks of age was subsequently followed by alterations in the innervation patterns of the muscle that became apparent at the 30 week time point. While imaging at earlier time points revealed long axons that synapse along longitudinal sections of myofibers, later time points exhibited more branched nerve fibers that synapsed across multiple myofibers (Figure 5). These changes are significant in transgenic G93A-*SOD1*-GFP zebrafish beginning at 30 weeks of age, and may reflect compensatory reinnervation of myofibers as previously observed in transgenic ALS mice [24,42]. MN loss follows NMJ denervation and distal axonal degeneration in patients and rodent ALS models [3,42]; therefore, we also quantified MN numbers in transgenic G93A-*SOD1*-GFP zebrafish between 30-60 weeks of age. We observed an approximate 50% reduction in the number of large MN cell bodies in the ventral horn of transgenic G93A-*SOD1*-GFP zebrafish spinal cords at 40 weeks of age compared to age-matched controls (Figure 6). This coincides with the sequence of events in ALS rodent models, as MN loss is evident around 100 days in G93A-*SOD1* mice, relative to initial NMJ defects as early as 47 days, and death at 131 days [3]. A timeline summarizing the sequence of observed morphological alterations in our transgenic G93A-*SOD1*-GFP zebrafish is depicted in Figure 7.

This characterization of our novel transgenic G93A-*SOD1*-GFP zebrafish supports and builds upon the previous findings in transgenic G93R-*sod1* zebrafish [40]. The transgenic G93R-*sod1* zebrafish exhibit a small but significant reduction in NMJ innervation at 11 days post-fertilization (dpf) in transgenic larvae, and a more significant loss of NMJ innervation at 12 months of age [40]. While intermediate time points were not described, these findings coincide with our later observations of NMJ loss between 40-60 weeks of age, and reflect the fact that significant NMJ defects are evident as early as 20 weeks of age in our model. MN loss is also evident in both models, with our transgenic G93A-*SOD1* zebrafish exhibiting an approximate 50% reduction in MN numbers by 40 weeks of age, and the transgenic G93R-*sod1* zebrafish having a 38% reduction in MN numbers at end-stage disease. In addition, transgenic G93R-*sod1* zebrafish lines exhibit reductions in survival that become evident around 18-22 months of age [40]. While preliminary findings in our transgenic G93A-*SOD1*-GFP zebrafish did not reveal any noticeable differences in survival (data not shown), it is possible based on the findings in the other model that the 60 week time frame for the current study was too short to detect quantifiable differences, and studies are currently ongoing to determine when or if survival is affected in our model. Taken together, both models depict a sequence of events that supports the “dying-back” theory in ALS, where defects at the NMJ are the first insult and then lead to progressive distal axonal degeneration and subsequent MN loss [3,4,24,42].

This newly established ALS model provides an additional system that will supplement our understanding of ALS neuromuscular pathology in the context of disease progression. While we observe significant neuromuscular defects that reflect a characteristic distal-to-proximal ALS disease course, there are still unanswered questions that should be addressed to understand why the disease course is not as severe in zebrafish as it is in G93A-*SOD1* rodents, and why drastic locomotor deficits are not observed even in the context of MN loss. A possible explanation for the reduced disease severity could relate to the relative expression levels of mutant SOD1 in the transgenic models. Our data indicate that G93A-SOD1-GFP expression levels were approximately 64% that of the endogenous zebrafish wtSOD1 (Figure 2C), whereas transgenic G93A-SOD1 rodent models exhibit robust levels of overexpression [22]. It is also possible that the disease course is affected by the regenerative ability of zebrafish; while neuronal regeneration is not prevalent in human or rodent spinal cords, evidence of MN regeneration has been reported in zebrafish [58]. The observed alterations in NMJ innervation patterns (Figure 4) support the possibility of compensatory reinnervation, and although we observe a decrease in MN numbers in transgenic G93A-*SOD1*-GFP zebrafish, regenerative markers must be examined in spinal cords to understand what role regeneration plays in disease pathogenesis in this model. Non-cell autonomous effects including astrocytes and glial activation also have a significant impact on disease progression in rodent ALS models [1,2]; therefore, the contributions of these cell types to disease pathogenesis should also be investigated. The impact of G93A-SOD1-GFP expression on other neuronal populations is also of interest, although no effects were seen on the commissural axon of the Mauthner neuron in the hindbrain, on sensory neurons of the lateral line, or on Rohon-Beard sensory neurons in the transient model described by Lemmens et al [36]. Finally, a detailed analysis of MN axons using electron microscopy and IHC with advanced imaging techniques to comprehend the mechanisms of axonal degeneration throughout the disease course will provide important insight into the “dying-back” mechanisms of MN degeneration in ALS [3,4,24,42].

## Conclusions

In summary, the novel transgenic G93A-*SOD1*-GFP zebrafish exhibit defects at the NMJ around 20 weeks of age, subsequent alterations in innervation patterns at 30 weeks of age, and evidence of MN loss around 40 weeks of age (Figure 7). These neuromuscular findings support the utilization of zebrafish as a model organism to investigate early events in ALS disease pathogenesis for therapeutic development. Furthermore, the generation of transgenic ALS zebrafish models expressing other familial ALS mutations such as *TDP43* should be considered, as they have the potential to provide additional insight into the mechanisms of ALS onset and progression.

## Methods

### Zebrafish

All zebrafish (*Danio rerio*) were maintained and handled in compliance with national and local animal welfare bodies and utilized per approved protocols (University of Michigan's Institutional Animal Care and Use Committee protocol #09835). Embryos were collected after timed mating of adult AB strain zebrafish for microinjection to establish the transient ALS models or generate the transgenic ALS lines used in these studies. Transgenic HB9:mGFP zebrafish [28] which express GFP in MNs were used to confirm the MN identification approach utilized for MN quantification. The transgenic ALS zebrafish described in this manuscript are available by contacting jamedowl@med.umich.edu, or if there is sufficient interest we will provide the lines through the Zebrafish International Resource Center (ZIRC; http://zebrafish.org/zirc/home).

### Transient overexpression of G93A-SOD1 in zebrafish

RNA generated from pCS2+ vectors allows for efficient transient expression of proteins in zebrafish embryos. To generate transient ALS zebrafish models, full length clones to human *wtSOD1* and G93A-*SOD1*[13] were subcloned into pCS2+ followed by restriction digest to linearize the constructs for generation of RNA using the mMessage mMachine SP6 kit (Ambion). The pCS2-based zebrafish *igf-I* construct was provided by Dr. Cunming Duan for *igf-I* RNA generation [59]. mRNA was microinjected into the yolk sac of 1-4 cell embryos and embryos were incubated at 28^o^C in 0.5X E2 media until processing for IHC or western blotting as previously described [60,61]. For IHC, microinjected zebrafish embryos and UIC zebrafish embryos were deyolked at 30 hpf and fixed overnight in 4% PFA. Fixed embryos were washed with 1x PBS and blocked for 2 h at room temperature in 2% BSA, 0.5% Triton-X 100 and 0.05% DMSO before incubation with the SV2 primary antibody (1:200; Developmental Studies Hybridoma Bank) overnight at 4^o^C. Embryos were then washed and incubated with AlexaFluor-594-conjugated anti-mouse secondary antibody (1:250, Invitrogen) for 2 h at room temperature, washed, and mounted onto glass slides using ProLong-Gold antifade reagent (Invitrogen, Eugene, OR). Slides were blinded and imaged using a Nikon AZ100 microscope and NIS Elements BS software. The length from the tip of the motor axon to the point where it exits the spinal cord was measured for 5 axons per embryo, corresponding to axons 13-17 using NIS Elements BS software. A minimum of 30 embryos per condition over 3 independent experiments were assessed for each data set.

Western blotting using standard protocols [21,45] was utilized to confirm SOD1 expression and activation of downstream IGF-I pathways. Briefly, microinjected zebrafish embryos and UIC zebrafish embryos were dechorionated at 30 hpf and protein lysates were prepared in RIPA buffer (20 mM Tris, pH 7.4, 150 mM NaCl, 1 mM EDTA, 0.1% SDS, 1 mM Na deocycholate, 1% Triton X-100, 0.1 trypsin units/L aprotinin, 10 mg/ml leupeptin, and 50 mg/ml PMSF; 15 μl per embryo). Equal amounts of protein were loaded into each lane of a 12.5% or 15% polyacrylamide gel for analysis of phospho-Akt/phospho-MAPK or SOD1 expression, respectively, and transferred to nitrocellulose. Blots were incubated with primary antibodies overnight at 4^o^C followed by incubation with appropriate horseradish peroxidase-conjugated secondary antibodies (Santa Cruz Biotechnology, Santa Cruz, CA). The following antibodies were utilized: phospho-Akt (1:1000; Cell Signaling, Danvers, MA), phospho-p44/42 MAPK (Thr202/tyr204; 1:1000; Cell Signaling), SOD-100 (1:5000; Stressgen, Victoria, Canada), and GAPDH (1:5000; Chemicon, Temecula, CA). Results following enhanced chemiluminescence with LumiGlO plus Peroxide reagent (Cell Signaling) were detected using the BioRad ChemiDoc XRS imaging system with Quantity One analysis software. GAPDH was used to confirm equal protein loading.

### Generation of transgenic ALS zebrafish lines

Stable transgenic zebrafish expressing G93A-*SOD1*-GFP were generated using Tol2-mediated transgenesis [47]. Constructs were generated with the Gateway-based cloning system using Tol2-constructs kindly provided by Dr. Chi-Bin Chien. Final constructs utilized the pDEST destination vector including a CMV promoter element, G93A-*SOD1* and a 3’ EGFP-fusion element with an SV40 late polyadenylation signal [47]. cDNA (10 ng/μl) was microinjected into 1-4 cell zebrafish embryos as described above along with *Tol2 transposase* RNA (100 ng/μl) to facilitate Tol2-mediated random integration into the genome. P0 transgenic embryos were raised to maturity and screened for germline incorporation. Successful generation of transgenic lines was evident via GFP expression in F1 generation embryos, or alternatively, via PCR analysis of genomic DNA from F1 generation embryos. Briefly, genomic DNA was extracted from 24 hpf embryos following standard protocols [62] and PCR-screened for the presence of incorporated human *SOD1* cDNA using the following primers: forward 5’-TTCGAGCAGAAGGAAAGTAATGGA-3’, reverse 5’-ACATTGCCCAAGTCTCCAACATGC-3’. Characterization of established transgenic lines was performed on F2 generation zebrafish and compared to age-matched control AB strain zebrafish. Stable transgene expression throughout the zebrafish lifespan and across generations was confirmed by assessing GFP fluorescence at each time point. Furthermore, western blotting was utilized to assay G93A-SOD1-GFP protein expression levels in transgenic 24-48 hpf embryos according to the protocol detailed above. Results are representative of at least 3 independent experiments from 3 clutches and densitometry results were normalized to GAPDH to quantify protein expression levels using Quantity One analysis software.

### Zebrafish activity monitoring

Swimming behavior was assessed using the rapid detection Noldus Larvae Activity Monitoring System (http://www.noldus.com/site/dpc200711027). An infrared camera tracks fish movements over a defined time interval and movements are then converted into distance tracks for analysis according to multiple activity parameters using the system’s proprietary software package, EthoVision XT 6.0. The system accommodates various sized multi-well plates to allow for quick and easy quantification of movement throughout the lifespan of the transgenic zebrafish. For activity monitoring, AB (n = 7) and transgenic G93A-*SOD1*-GFP (n = 11) zebrafish were monitored weekly between the ages of 10 and 30 weeks and every other week between 30 and 70 weeks of age, for 10 minutes to record spontaneous swimming behavior. Results were analyzed for average swimming velocity and time resting during the 10 minute monitoring interval.

### Neuromuscular characterization

MN axonal and NMJ defects and degeneration were examined by IHC per standard protocols [45,52,55]. Briefly, adult AB and transgenic G93A-*SOD1*-GFP zebrafish were euthanized at 10, 20, 30, 40, and 60 weeks of age. Fish were fixed overnight in 2% PFA, cryoprotected in PBS containing 30% sucrose and muscle tissue was dissected and embedded in OCT for sectioning and IHC. Longitudinal sections (20 μm) were obtained on a Leica cryostat and then processed for IHC using our previously published method [60,63]. Briefly, sections were blocked for 30 minutes with 5% normal goat serum / 0.1% triton-X100 / 1x PBS and incubated with AlexaFluor 647-conjugated αBTX (1:1000; Molecular Probes, Invitrogen, Carlsbad) for 30 minutes to label postsynaptic AChRs. Sections were then washed and incubated overnight with SV2 (1:200; Hybridoma Bank, Iowa City, IA) and NF (1:1000; Chemicon) primary antibodies to label MN axons. After washing, Alexa Fluor 594-conjugated IgG secondary antibody (Molecular Probes) was applied for 2 h, followed by rinsing and mounting in Prolong Gold antifade reagent with DAPI (Invitrogen). Slides were blinded for z-stack imaging with a Zeiss Apotome or a Nikon A1 laser scanning confocal system with TiE microscope. Imaging was done at the University of Michigan Microscopy & Image Analysis Core (http://www.med.umich.edu/mil/index.htm) or the Morphology and Image Analysis Core of the Michigan Diabetes Research and Training Center (http://www.med.umich.edu/mdrtc/cores/MIACCore/index.html), respectively.

Differences in NMJ integrity were determined by examining SV2/NF and αBTX co-localization to calculate the percent of innervated junctions and by examining NMJ innervation patterns. The percent of innervated synapses was determined by calculating the number of AChR clusters fully or partially innervated by nerve terminals as previously described using MetaMorph (version 7.4.4, Molecular Devices, Sunnyvale, CA) [40,45,50,64,65]. At least 3 random images per slide were acquired from 3 zebrafish per time point, for a minimum of 9 images per line. Elements software was used to visualize orthogonal XZ and YZ views of the z-seried to confirm colocalization. Qualitative assessment of innervation patterns in the muscle consisted of assessing branching patterns of individual fibers for at least 3 images per slide from 3 zebrafish per time point per condition. Innervation patterns were scored as long, moderately branched or complex. Long axons (score of 1) are axons that do not exhibit a major branch point along the length of visible axonal segment. Moderately branched axons are nerve fibers that exhibit a single major branch point (score of 2) or two branch points (score of 3). Complex innervation patterns consist of segments with three or more major branches (score of 4+).

### Motor neuron quantification

MN loss was determined by counting the total number of MNs in spinal cord sections [66]. Adult AB and transgenic ALS zebrafish at 30, 40, and 60 weeks of age were fixed and cryoprotected and spinal cords were dissected and prepared for sectioning as described above for muscle tissue. MNs in 20 μm cross-sections were labeled with Cresyl violet Nissl stain (Catalog # C-3886; Sigma, St. Louis, MO) as previously described [67]. Briefly, sections were stained with Cresyl violet and cleared and dehydrated prior to mounting with DPX. Images were acquired using a Nikon Microphot-FXA microscope and the number of spinal MNs per spinal cord ventral horn was quantified for multiple sections throughout the spinal cord from at least 3 fish per line at each time point. The approach identifying MNs by cell body size and localization to the ventral horn was validated using transgenic HB9:mGFP zebrafish.

### Statistical analysis

All results are representative of at least 3 independent experiments and slides were blinded prior to imaging and analysis. Multiple independent transgenic ALS zebrafish lines were generated to minimize any positional effects of the transgene insertion. Statistical significance was determined using one-way ANOVA followed by Tukey’s multiple comparison test or linear regression analysis (GraphPad Prism).

## Abbreviations

αBTX: α-bungarotoxin; AChR: Acetylcholine receptor; ALS: Amyotrophic lateral sclerosis; Als2: Alsin; dpf: Days post-fertilization; fALS: Familial amyotrophic lateral sclerosis; FUS: Fused in sarcoma; hpf: Hours post-fertilization; IGF-I: Insulin-like growth factor-I; IHC: Immunohistochemistry; MAPK: Mitogen activated protein kinase; MN: Motor neuron; NF: Neurofilament; NMJ: Neuromuscular junction; SOD1: Cu^2+^/Zn^2+^ superoxide dismutase; TDP43: TAR DNA binding protein 43; UIC: Uninjected control; wtSOD1: Wild-type Cu^2+^/Zn^2+^ superoxide dismutase; ZIRC: Zebrafish international resource center.

## Competing interests

The authors have no competing or financial interests to declare.

## Authors’ contributions

SAS initiated and designed the study, made the transgenic lines, and designed and performed the experiments described in this manuscript. ASB assisted with all experiments and managed the zebrafish facility. GZ-B, MP, AAR, and SGP were involved in the transient ALS model experiments. SSO contributed to the transgenic zebrafish experiments. Data analysis was performed by SAS and JSL. SAS wrote the manuscript. JSL, JJD, and ELF provided critical feedback on experimental design and edited the manuscript. All authors read and approved the final manuscript.
